# Efeito do Diterpeno Manool sobre a Pressão Arterial e Reatividade Vascular em Ratos Normotensos e Hipertensos

**DOI:** 10.36660/abc.20190198

**Published:** 2020-10-13

**Authors:** Ariadne Santana e Neves Monteiro, Debora Ribeiro Campos, Agnes Afrodite Sumarelli Albuquerque, Paulo Roberto Barbosa Evora, Luciana Garros Ferreira, Andrea Carla Celotto

**Affiliations:** 1 Universidade de São Paulo Faculdade de Medicina de Ribeirão Preto Ribeirão PretoSP Brasil Universidade de São Paulo Faculdade de Medicina de Ribeirão Preto, Ribeirão Preto, SP – Brasil

**Keywords:** Doenças Cardiovasculares, Hipertensão, Diterpeno, Manool, Reatividade, Plantas Medicinais, Óxido Nítrico, Ratos

## Abstract

**Fundamento:**

Diversos estudos têm mostrado que as classes de diterpenos exercem efeito significativo no sistema cardiovascular. Os diterpenos, em particular, estão entre os principais compostos associados às propriedades cardiovasculares, como a propriedade vasorrelaxante, inotrópica, diurética e a atividade hipotensora. Embora o mecanismo de vasorrelaxamento do manool seja visível, seu efeito sobre a pressão arterial (PA) ainda é desconhecido.

**Objetivo:**

Avaliar o efeito hipotensor in vivo do manool e verificar o efeito de vasorrelaxamento ex vivo em anéis aórticos de ratos.

**Métodos:**

Os animais foram divididos aleatoriamente em dois grupos: normotensos e hipertensos. O grupo normotenso foi submetido à cirurgia sham e adotou-se o modelo 2R1C para o grupo hipertenso. Realizou-se monitoramento invasivo da PA para testes com manool em diferentes doses (10, 20 e 40 mg/kg). Foram obtidas curvas de concentração-resposta para o manool nos anéis aórticos, com endotélio pré-contraído com fenilefrina (Phe) após incubação com Nω-nitro-L-arginina metil éster (L-NAME) ou oxadiazolo[4,3-a]quinoxalina-1-ona (ODQ). Os níveis plasmáticos de óxido nítrico (NOx) foram medidos por ensaio de quimioluminescência.

**Resultados:**

Após a administração de manool, a PA se reduziu nos grupos normotenso e hipertenso, e esse efeito foi inibido pelo L-NAME em animais hipertensos apenas na dose de 10 mg/kg. O manool ex vivo promoveu vasorrelaxamento, inibido pela incubação de L-NAME e ODQ ou remoção do endotélio. Os níveis plasmáticos de NOx aumentaram no grupo hipertenso após a administração de manool. O manool induz o relaxamento vascular dependente do endotélio na aorta de ratos, mediado pela via de sinalização NO/cGMP e redução da PA, e também pelo aumento plasmático de NOx. Esses efeitos combinados podem estar envolvidos na modulação da resistência periférica, contribuindo para o efeito anti-hipertensivo do diterpeno.

**Conclusão:**

Esses efeitos em conjunto podem estar envolvidos na modulação da resistência periférica, contribuindo para o efeito anti-hipertensivo do diterpeno.

## Introdução

Os diterpenos são uma ampla classe de metabólitos químicos, amplamente distribuídos no reino vegetal, com mais de 12.000 compostos conhecidos.^1^^,^^2^ Eles podem ser divididos em dois tipos: diterpenos de metabolismo especializado (secundário) e diterpenos de metabolismo geral (primário). Os diterpenos secundários podem ter funções nas interações ecológicas das plantas com outros organismos e benefícios em fármacos, perfumes, resinas e outros bioprodutos industriais com grande relevância econômica.^1^^,^^2^ Diversos metabólitos secundários, como terpenos, ácidos fenólicos, polifenóis, flavonoides e antocianinas, foram relatados em espécies de sálvia. Essas espécies são vistas como excelentes fontes de diterpenos.^3^ De acordo com os achados quimiotaxonômicos, o manool foi relatado anteriormente nas seguintes espécies de sálvia: *S. sclarea, S. pubescens, S. lavandulifolia, S. hypoleuca*, *S. miltiorrhizae*. Também está presente em outras espécies, como na *Pinuscaribaea (Pinaceae), Lourteigiastoechadifolia (Asteraceae)* e *Halocarpusbiformis (Podocarpaceae)*. No entanto, o manool é o principal diterpeno das várias espécies de sálvia, sendo encontrado em maior concentração na *Salvia officinalis.*^4^

A biossíntese das unidades estruturais de isopreno de uma ampla variedade de terpenos, incluindo os diterpenos, ocorre pela via da desoxilulose. Essa via aumenta e evolui para dois produtos distintos: isopentenildifosfato (IPP) e dimetilalildifosfato (DMAPP). Mais especificamente, o manool, cuja composição química é C_20_H_34_O, é um diperteno do tipo labdano bicíclico. Sua estrutura se baseia em um esqueleto carbonado do tipo 2E, 6E, 10E-geranilgeranilpirofosfato (GGPP).^5^^–^^7^

A descoberta de novas substâncias com atividade anti-hipertensiva, baixo custo e poucos efeitos adversos é ainda um aspecto desejável e de importância para a utilização clínica.^8^ Porém, várias dificuldades são encontradas para esse fim, como a escolha do modelo experimental, obtenção de extratos padronizados e a dificuldade de obtenção, isolamento e identificação das substâncias ativas.^9^^,^^10^ A opção de conduzir pesquisas, a partir da indicação de plantas utilizadas pelas comunidades, encurta o percurso de desenvolvimento de um novo fármaco, pois os pesquisadores dispõem, antes mesmo de se iniciarem estudos científicos, de uma indicação de qual atividade biológica esta droga poderia apresentar.^11^^,^^12^

Os diterpenos, em particular, estão entre os principais compostos com ligação às propriedades cardiovasculares, tais como vasorrelaxante, inotrópica, diurética e hipotensiva. A ação vascular exercida por esses compostos parece envolver múltiplos mecanismos, como endotélio dependente e endotélio independente, aumento de prostaciclina e bloqueio de canais de cálcio dependentes de voltagem.^13^^–^^17^

**Figura 1:**
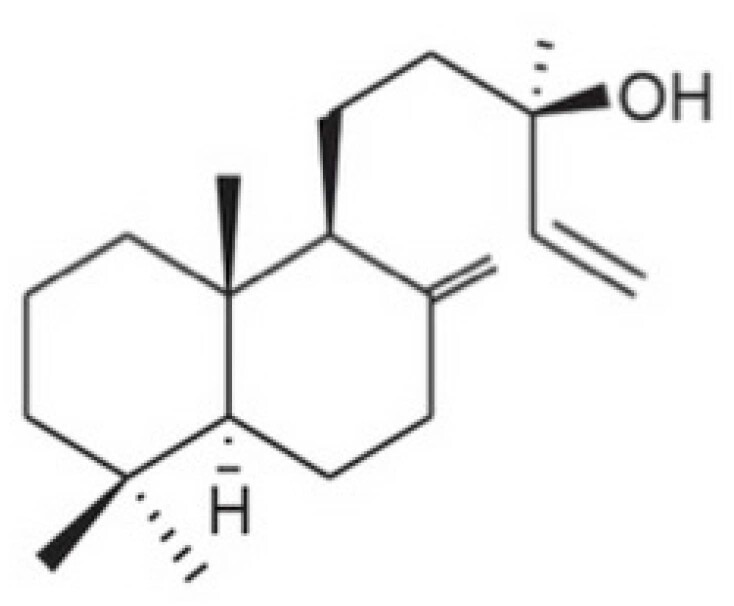
Estrutura química do Manool.^10^^,^^11^

Conforme descrito anteriormente na revisão da literatura, o manool — C_20_H_34_O — é um diterpeno do tipo labdano, comumente encontrado em diversas famílias de plantas, é o principal diterpeno de várias espécies de sálvia, e está presente em concentrações mais elevadas na *Salvia officinalis* (Figura 1).^1^^,^^3^^,^^18^^,^^19^ É uma espécie da família *Lamiaceae (Labiateae)*, originária do sul da Europa. Apresenta hábito de crescimento herbáceo ou arbustivo de pequeno porte, é planta perene que floresce no Hemisfério Sul entre os meses de agosto e dezembro.^20^

Li et al.,^21^ constaram que embora o manool possua atividades desconhecidas do ponto de vista cardiovascular, ele deve ser considerado como fator crucial nos estudos a serem realizados. Além disso, pode ser visto como um novo condutor para o tratamento de doenças cardíacas, merecendo mais investigação.^4^^,^^21^^,^^22^ O protocolo experimental incluiu observações sobre os níveis plasmáticos de óxido nítrico (NOx) em animais hipertensos e o impacto da manool na BP de animais após a administração de diferentes doses do composto.

Sabendo que o manool pertence à classe dos compostos diterpênicos, com potencial uso no tratamento da hipertensão, o presente trabalho teve como objetivo avaliar o possível efeito vasodilatador e os mecanismos celulares envolvidos na resposta de relaxamento de anéis aórticos de ratos. Portanto, o objetivo foi avaliar o efeito hipotensor in vivo do manool e verificar o efeito vasorrelaxamento ex vivo em anéis aórticos de ratos.

## Materiais e Métodos

### Declaração de Ética e Animais

As políticas de manejo de animais e procedimentos experimentais foram analisadas e aprovadas pela Comissão de Ética em Experimentação Animal da Faculdade de Medicina de Ribeirão Preto da Universidade de São Paulo (n. 060/210), seguindo as orientações da Diretiva 2010/63 UE da Comissão Europeia. Trinta e quatro ratos Wistar machos (180–220 g) foram acondicionados em condições laboratoriais padrão (ciclo claro/escuro de 12 horas a 21 °C) com livre acesso à água e ração. Os animais foram divididos aleatoriamente em cinco grupos de 7 animais para protocolos de pressão arterial normotensa e hipertensa (veículo normotenso, manool normotenso; veículo hipertenso, manool hipertenso e manool hipertenso + L-NAME). Os animais alocados nos grupos normotensos foram sham-operados, enquanto os animais alocados nos grupos hipertensos foram submetidos ao procedimento cirúrgico 2R1C (dois rins-um-clipe hipertensos) para indução da hipertensão. Utilizou-se um outro grupo de 6 animais que não realizaram nenhum procedimento (intactos) para estudos de reatividade vascular ex vivo.

### Fármacos

Manool, acetilcolina (ACh), 1H-[1,2,4]oxadiazolo[4,3-a]quinoxalina-1-ona (ODQ) e fenilefrina (Phe), da Sigma Chemical Company (St. Louis, MO, EUA); éster metílico de Nω-nitro-L-arginina (L-NAME), obtido na Calbiochem (San Diego, CA, EUA); Vetec Química Fina Ltda forneceu isoflurano da Abbott e todos os sais usados para a preparação da solução de Krebs. Quase todos os fármacos foram preparados com água destilada, sendo o manool solubilizada em dimetilsulfóxido (50 uL) e diluído em etanol/água (2:10, volume total 200 uL). Para os experimentos de reatividade vascular, 100 uL foram diluídos em 900 uL de água, formando o estoque (10^−3^). A partir desse estoque, preparou-se a curva. O volume usado a partir dessa curva foi de 10 uL em uma cuba de 10 ml. Portanto, após tantas diluições, o veículo não promove nenhum efeito na reatividade vascular.

### Indução da Hipertensão

Após anestesia intraperitoneal com cetamina (50 mg/kg) e xilazina (10 mg/kg), a artéria renal foi exposta. Os grupos hipertensos apresentaram constrição parcial da artéria renal esquerda principal com clipe de prata com abertura de 0,10 mm (2R1C), enquanto os grupos normotensos tiveram a artéria renal esquerda principal isolada, mas não receberam o clipe (sham). Para monitorar o desenvolvimento da hipertensão, a pressão arterial sistólica (PAS) foi medida de forma não invasiva por meio da pletismografia de cauda, uma vez por semana. (Kent Scientific Corporation, Connecticut, EUA). Os ratos 2R1C foram considerados hipertensos com PAS de cauda ≥ 160 mmHg na 3ª semana após os procedimentos cirúrgicos. Os ratos 2R1C com PAS <160 mmHg na 3ª semana foram eutanasiados. Menos de 10% dos animais apresentaram PAS <160 mmHg. Os ratos que foram sham-operados foram incluídos no grupo normotenso.

### Efeito do Manool na Pressão Arterial

Três semanas após a indução da hipertensão, os animais foram anestesiados, e a artéria e veia femoral foram canuladas, respectivamente, para medição contínua da pressão arterial sistólica (PAS) e administração de medicamentos. Após anestesia (uretano, 2 mg/kg, intraperitoneal), canulação vascular e período de estabilização (20 minutos) com registro contínuo da pressão arterial sistólica (PAS) em tempo real, três doses de manool (10, 20 e 40 mg/kg) ou veículo (dimetilsulfóxido — DMSO — e água+etanol) foram administrados aos ratos normotensos e hipertensos. Cada dose foi administrada em bolus intravenoso de 200 µL e o intervalo entre cada dose consecutiva foi de 6 minutos. Os animais que receberam veículo não receberam manool. Para cada animal, a variação na pressão arterial sistólica (ΔPAS) foi calculada subtraindo a média dos valores mais baixos de PAS imediatamente após a administração de manool da média dos valores basais de PAS antes do manool ou bolus do veículo. A pressão arterial média foi medida por meio do MP System 100 A (BioPac System, Inc., Santa Bárbara, CA, EUA).

### Reatividade Vascular

Os experimentos foram realizados em anéis aórticos de ratos normotensos. Seis ratos Wistar machos (280–300 g) foram anestesiados com isoflurano inalatório, seguido de exsanguinação da aorta abdominal e toracotomia para retirada da aorta torácica. A aorta torácica foi cuidadosamente dissecada, confirmada como livre de tecido conjuntivo e imediatamente imersa em solução de Krebs. A solução de Krebs era composta por NaCl (118,0 mM), KCl (4,7 mM), CaCl2 (2,5 mM), KH2PO4 (1,2 mM), MgSO4 (1,66 mM), glicose (11,1 mM) e NaHCO3 (25,0 mM); a solução tinha pH 7,4. A aorta torácica imersa em solução de Krebs foi cortada em anéis de 4 a 5 mm de comprimento. Para os testes, o anel com endotélio desnudado foi removido esfregando-se suavemente o vaso da superfície interna com uma haste de aço fina. Esse procedimento remove efetivamente o endotélio, mas não afeta a capacidade do músculo liso vascular de se contrair ou relaxar. Os anéis aórticos foram colocados em 10 mL de banho orgânico para tecido isolado contendo solução de Krebs, a 37 °C, e 95% O_2_/5% CO_2_ (pH 7,4) para medir a força isométrica por meio do equipamento Grass FT03 (Grass Instrument Company, Quincy, MA, EUA). Cada anel foi alongado até o ponto ótimo de estiramento-tensão de 2,0 g, determinado em um estudo piloto, e permaneceram sob esta tensão por 60 min. Durante esse tempo, os tecidos foram lavados a cada 15 minutos. O endotélio foi considerado presente (E+) registrando-se o relaxamento de 80% induzido por Ach (10^−6^ M) após a pré-contração com Phe (10^−7^ M). O endotélio foi considerado ausente (E−) quando a resposta de relaxamento não ocorreu. Em seguida, cada anel foi lavado e reestabilizado por 30 min. Os anéis aórticos foram contraídos com Phe (10^−7^ M) depois que um platô estável foi atingido e as curvas de dose-resposta de manool foram obtidas. Os ensaios de concentração-resposta nos banhos orgânicos foram realizados na presença ou ausência de: L-NAME (2x10^−4^ M), um inibidor não específico da óxido nítrico sintase e ODQ (10^−4^ M), um inibidor da guanililciclase.^20^ As preparações foram incubadas com os inibidores por 30 min. Não realizamos curvas de dose-resposta com um veículo porque a diluição foi realizada em água. A solução inicial 1 M (50 uL de DMSO + 30 uL de etanol + 120 uL de água) sofreu diluição em série para 10^−1^ M em água.

### Medições Plasmáticas Indiretas de NO

Coletou-se amostras de sangue (1 ml) da artéria femoral após a última curva dose-resposta em veículo normotenso e manool hipertenso, sendo colocadas em tubos heparinizados. Após centrifugação do sangue (3000×g, 10 minutos, 4 °C), o plasma foi imediatamente imerso em nitrogênio líquido e mantido a −70 °C até a dosagem de nitrito e nitrato (NOx). As amostras foram analisadas em duplicata para NOx por ensaio de quimioluminescência à base de ozônio. As amostras plasmáticas foram brevemente tratadas com etanol frio (1 volume de plasma: 2 volumes de etanol por 30 minutos a −20 °C) e centrifugadas (4000×g, 10 minutos). Os níveis de NOx foram medidos pela injeção de 25 *μ*L de sobrenadante em recipiente de purga de vidro contendo 0,8% de vanádio (III) em HCl (1 N) a 90 °C, que reduz o NOx a óxido nitroso. Uma corrente de nitrogênio foi borbulhada através do recipiente de purga contendo vanádio (III), em seguida através de NaOH (1 N), e então em um analisador de NO (Sievers® Nitric Oxide Analyzer 280, GE Analytical Instruments, Boulder, CO, EUA).

### Análise Estatística

Os dados são apresentados como média ± erro-padrão da média. Realizamos análises estatísticas com o teste T de Student, análise de variância simples (ANOVA), pós-teste de Bonferroni, e ANOVA de duas vias com medidas repetidas, com o pós-teste de Bonferroni para detectar possíveis diferenças entre os valores do estudo. Para cada figura, a legenda descreve qual teste foi realizado para análise. Considerou-se significativo um p<0,05 (Prism 5.0, GraphPad Software, San Diego, CA, EUA). Um tamanho de amostra de (N = 5–7) por grupo forneceu 95% de poder com um nível de significância de 0,05% em protocolos de medição da pressão arterial in vivo. Além disso, um tamanho de amostra de (N = 6–8) animais por grupo forneceu 95% de poder com um nível de significância de 0,05 para detectar uma redução relativa de 10% na contração máxima em vasos pré-contraídos. O número de animais foi escolhido com base na literatura.^20^^,^^23^^,^^24^

## Resultados

**Figura 2:**
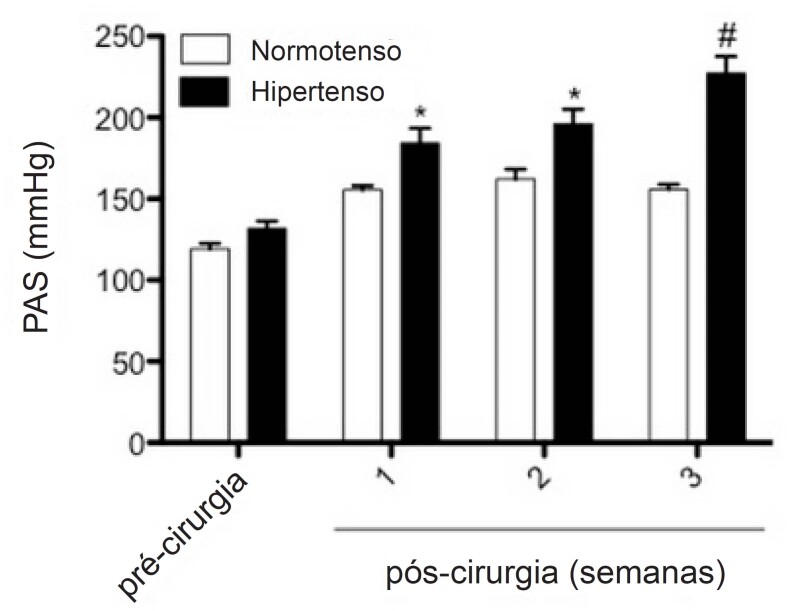
Evolução temporal da pressão arterial sistólica (PAS) de forma não invasiva em animais normotensos e hipertensos. Os valores representam a média ± erro padrão da pressão arterial média antes da cirurgia de colocação de clipe (pré-operatório) e três semanas após a cirurgia. *p<0,05 e # p<0,01 indicam diferença significativa entre o grupo de hipertensos e normotensos. ANOVA de duas vias, pós-teste de Bonferroni. n=14 normotenso e n=14 hipertenso.

Antes dos procedimentos cirúrgicos, não havia diferenças na PA entre os grupos normotenso e hipertenso. Porém, após a indução da hipertensão, da 1ª à 3ª semana, a PA mostrou-se significativamente mais elevada nos ratos hipertensos (130,6 mmHg versus 193,0 mmHg) (Figura 2).

A avaliação do peso corporal mostrou que, na primeira semana, os grupos apresentavam cargas semelhantes. Porém, ao final de três semanas, o grupo de hipertensos apresentou valores significativamente menores em relação ao grupo de normotensos (Tabela 1).

**Tabela 1:** Evolução temporal do peso corporal de animais normotensos e hipertensos

Evolução do peso corporal (g)		
Grupos	Inicial	Final
Normotenso	233,4±7,1	480,2±10,2
Hipertenso	239,4±7,7	404,8±18,2*

Cada valor representa a média ± erro-padrão da média.

* p<0,05 indica diferença significativa entre o grupo hipertenso e o grupo normotenso. Teste t de Student.

**Figura 3:**
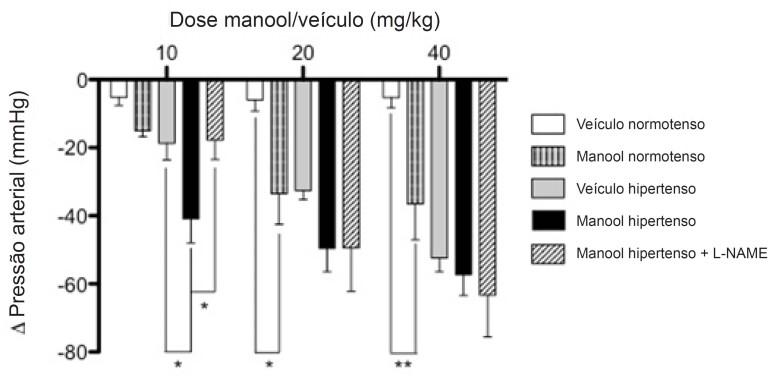
Alteração da pressão arterial sistólica (ΔPAS) após administração de manool ou veículo em ratos normotensos e hipertensos. Os dados são apresentados como média ± erro-padrão da média. Veículo normotenso (n=7), manool normotenso (n=7), veículo hipertenso (n=7), manool hipertenso (n=7) e manool hipertenso + L-NAME (n=7), *p<0,05, ** p<0,01 indica diferença significativa. ANOVA de duas vias, pós-teste de Bonferroni.

Na análise da PAS in vivo, apenas a cirurgia (2R1C) foi capaz de alterar o sangue (veículo normotenso versus veículo hipertenso). O manool promoveu resposta dose-dependente na PAS, reduzindo significativamente a pressão a partir da dose de 20 mg/kg no grupo normotenso, não havendo diferença entre 20 e 40 mg/kg neste grupo para o manool. No grupo hipertenso, apenas uma dose menor de manool (10 mg/kg) reduziu a PAS em comparação ao grupo controle (veículo hipertenso), e a administração prévia de L-NAME preveniu o efeito manool. No grupo hipertenso, o efeito manool não foi dose-dependente (Figura 3).

**Figura 4:**
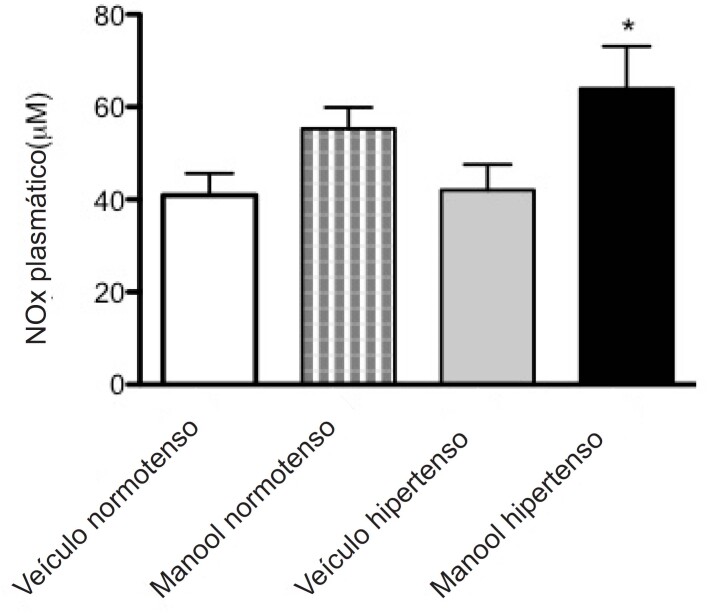
Níveis plasmáticos de nitrito e nitrato (NOx) em veículos normotensos e manool e veículos hipertensos e animais com manool. ANOVA de uma via, pós-teste de Bonferroni (n=7). *p<0,01 indica diferença significativa entre veículo hipertenso e manool hipertenso.

O NOx plasmático fica um pouco alto no grupo normotenso após a administração de manool, mas não é significativo. Porém, no grupo hipertenso, o manool promoveu aumento nos níveis plasmáticos de NOx (Figura 4).

**Figura 5:**
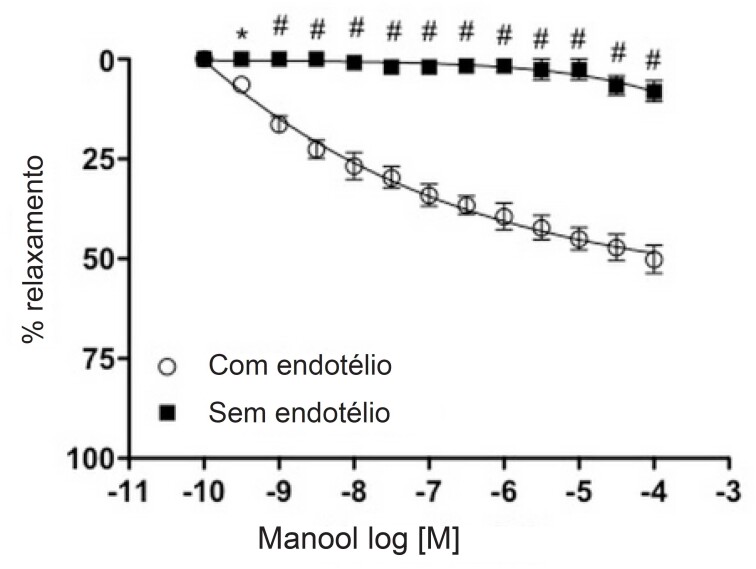
Curva de relaxamento em anéis de aorta torácica de ratos com endotélio intacto e endotélio desnudado expostos a manool. Os anéis foram pré-contraídos com fenilefrina (Phe) (10-7.M). Todos os valores correspondem à média ± erro-padrão da média (n=6). *p<0,05 e # p<0,001. ANOVA de duas vias com medidas repetidas e pós-teste de Bonferroni.

**Figura 6:**
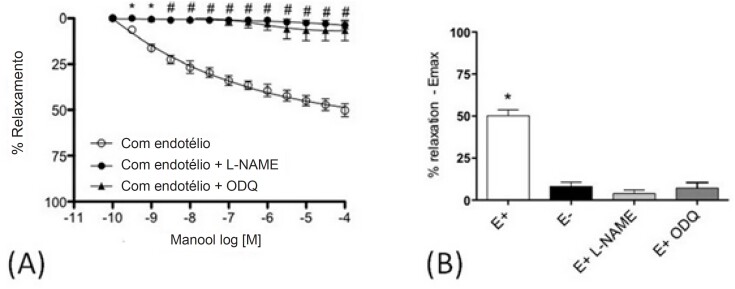
Curva de relaxamento em anéis de aorta torácica de ratos com endotélio intacto expostos a manool na presença e ausência de L-NAME (2x10-4 M) ou oxadiazolo[4,3-a]quinoxalina-1-ona (ODQ) (10-4 M). (A) curva dose-resposta e (B) Gráfico de barras Emax. Os anéis foram pré-contraídos com fenilefrina (Phe) (10-7.M). Todos os valores correspondem à média ± erro-padrão da média (n=6). * p<0,05 e #p<0,001 indicam diferenças significativas entre cada grupo e o grupo controle (vasos com endotélio); ANOVA de duas vias com medidas repetidas e pós-teste de Bonferroni.

Sobre os experimentos de reatividade vascular, o manool promoveu um relaxamento dose-dependente em anéis intactos (Figura 5), pré-contraídos com Phe. A incubação com L-NAME ou ODQ bloqueou o relaxamento induzido por manool em anéis com endotélio intacto da mesma forma que a remoção do endotélio (Figuras 6A e 6B).

## Discussão

Pesquisas anteriores mostraram que diterpenos labdanos têm uma ampla gama de efeitos farmacológicos, como a capacidade de inibir a replicação do vírus HIV, prevenir resfriados comuns, ação antimalárica, antibacteriana, anti-inflamatória, anti-hiperglicêmica, evitar a disenteria, além de suprimir diversas células cancerosas.^6^^,^^13^ Já em aspecto cardiovascular, evidenciaram: significativa redução de estenose em artérias ateroscleróticas, associada à redução das taxas de reestenose após angioplastia em coelhos; redução da agregação plaquetária in vitro e ação anti-hipertensiva em ratos.^13^^,^^15^^–^^17^^,^^25^ São, portanto, vistos como fonte promissora de novos protótipos para a descoberta e desenvolvimento de novos agentes terapêuticos cardiovasculares.

Os diterpenos, em particular, estão entre os principais compostos com ligação às propriedades cardiovasculares, como a propriedade vasorrelaxante, inotrópica, diurética e atividade hipotensora.^26^ A ação vascular exercida por esses compostos parece envolver múltiplos mecanismos, como endotélio dependente e endotélio independente, aumento de prostaciclinas e bloqueio de canais de cálcio dependentes de voltagem.

No presente estudo, utilizamos o modelo 2R1C para investigar o possível efeito anti-hipertensivo do manool. Esse modelo produziu resultados satisfatórios para a indução de hipertensão, com aumento significativo da pressão arterial em animais, após três semanas da cirurgia. Mesmo na primeira semana após a cirurgia, a PAS de 2R1C foi maior que em um animal normotenso. A PAS encontrada em animais hipertensos está de acordo com outros autores que avaliaram um modelo semelhante.^23^^,^^27^^,^^28^

Os resultados obtidos após a administração de 3 doses crescentes de manool mostraram que este composto foi capaz de reduzir a PA em ratos normotensos e hipertensos. Em animais normotensos, o manool apresenta efeito dose-resposta positivo. Esse achado difere de outros compostos naturais, incluindo o ácido rosmarínico, que reduziu a PA apenas em animais hipertensos.^23^ Esse perfil de resposta não é observado em animais hipertensos, onde o aumento da dose não representa um efeito mais significativo. A ΔPAS é a mesma após 10, 20 e 40 mg/kg de manool em animais hipertensos; em outras palavras, independentemente das doses, a pressão arterial máxima era de cerca de 40–50 mmHg. Porém, como no grupo com veículo hipertenso houve redução da PAS, apenas 10 mg/kg foi capaz de reduzir efetivamente a pressão.

Nossa hipótese para esse efeito anti-hipertensivo da manool baseou-se em estudos recentes sobre a atividade vasodilatadora de diterpenos mediada por NO.^13^^,^^15^^,^^16^^,^^26^ Demonstrou-se que a hipertensão tem forte associação com a formação de espécies reativas de oxigênio (EROs).^29^ Consequentemente, a inativação do NO pelo superóxido induz o desenvolvimento de disfunção endotelial em doenças cardiovasculares.^30^ A propriedade de alguns compostos de aumentar o NO pode ser atraente para reduzir a disfunção endotelial da hipertensão. Nossos achados indicam que o efeito anti-hipertensivo do manool pode ser parcialmente mediado pelo NO, uma vez que a administração de L-NAME antes da injeção de manool bloqueia a redução da PAS em animais hipertensos apenas na dose de 10 mg/kg. Corroborando esses achados, a concentração plasmática de NOx aumentou significativamente apenas nos animais hipertensos que receberam manool. Alguns estudos de NOx no modelo 2R1C mostram que a hipertensão pode reduzir esses níveis, mas nosso achado está em desacordo com esses dados, talvez por causa do tempo da cirurgia 2R1C.^31^^,^^32^ Embora o efeito anti-hipertensivo total do manool permaneça desconhecido, outras hipóteses podem ser levantadas, como inibição e modulação da ECA (enzima de conversão da angiotensina).^33^ Demonstrou-se que, no modelo 2R1C, há aumento na atividade da ECA plasmática e alguns peptídeos naturais de arroz, terpenos, fitoestrogênio e compostos polifenólicos podem reduzir a atividade da ECA,^20^^,^^34^^,^^35^ o que poderia caracterizar esse mecanismo como complementar ao NO na manutenção da PA.

Seria possível atribuir o efeito anti-hipertensivo do manool a um efeito direto na reatividade vascular que não inclui o aumento do NO sistêmico. O presente estudo mostrou que o manool induz o relaxamento aórtico em ratos apenas na presença de endotélio e pré-incubação dos anéis aórticos com inibidores da sintase de óxido nítrico (NOS) ou guanilato ciclase (GC). As propriedades cardiovasculares do diterpeno estão relacionadas ao bloqueio dos canais de Ca^2+^ e ativação de NO/GMPc (guanosina monofosfato cíclica).^13^ O endotélio produz vasodilatadores potentes, como o fator relaxante derivado do endotélio (EDRF, NO), prostaciclina e fator de hiperpolarização derivado do endotélio (EDHF). O NO é o mediador predominante na condutância e nas grandes artérias, enquanto o EDHF e a prostaciclina são mais prevalentes nas artérias menores, como os vasos mesentéricos, artérias coronárias e vasos de resistência periférica.^36^ Corroborando nossos achados, alguns têm-se relatos de que alguns diterpenos, como o 14-desoxi-11,12-dihydroandrographolide e 14-desoxyandrographolide dilatam anéis aórticos. O composto 14-desoxi-11,12-dihydroandrographolide teve um efeito hipotensor em ratos anestesiados. Ambos os compostos exercem sua atividade vasorrelaxante pela liberação de NO e ativação da via da guanilateciclase, bem como pelo bloqueio do influxo de Ca^2+^ por meio de canais de Ca^2+^ operados por voltagem e por receptor.^13^^,^^37^^–^^39^ No presente estudo, também sugerimos que o manool tem um efeito vasorrelaxante dependente do endotélio operando através da via NO/GMPc.

## Conclusão

Em resumo, o manool induz relaxamento vascular dependente do endotélio na aorta de ratos mediado pela via de sinalização NO/GMPc e redução da PA também pelo aumento plasmático de NOx. Esses efeitos em conjunto podem estar envolvidos na modulação da resistência periférica, contribuindo para o efeito anti-hipertensivo desse diterpeno.
