# Supplementary material for: Efeito do Diterpeno Manool sobre a Pressão Arterial e Reatividade Vascular em Ratos Normotensos e Hipertensos
Source: Arq Bras Cardiol. 2020 Oct 13;115(4):669–77. [Article in Portuguese] doi: 10.36660/abc.20190198 (PMC8386962; doi:10.36660/abc.20190198)
Supplement: Translation

# Translation: Effect of Diterpene Manool on the Arterial Blood Pressure and Vascular Reactivity in Normotensive and Hypertensive Rats

**Authors:** Ariadne Santana e Neves Monteiro, Debora Ribeiro Campos, Agnes Afrodite Sumarelli Albuquerque, Paulo Roberto Barbosa Evora, Luciana Garros Ferreira, Andrea Carla Celotto

**Affiliations:** 1 Ribeirão PretoSP Brazil Universidade de São Paulo Faculdade de Medicina de Ribeirão Preto, Ribeirão Preto, SP – Brazil

**Keywords:** Cardiovascular Diseases, Hypertension, Diterpenes, Manool, Reactivity, Nitric Oxide, Rats

## Abstract

**Background:**

Many studies have shown that the diterpenoid classes exert a significant effect on the cardiovascular system. Diterpenes, in particular, are among the main compound links to cardiovascular properties such as vasorelaxant, inotropic, diuretic and hypotensive activity. While the manool vasorelaxation mechanism is visible, its effect on blood pressure (BP) is still unknown.

**Objective:**

To evaluate the in vivo hypotensive effect of manool and check the ex vivo vasorelaxation effect in rat aortic rings.

**Methods:**

The animals were divided randomly into two groups: normotensive and hypertensive. The normotensive group was sham-operated, and the 2K1C model was adopted for the hypertensive group. Invasive BP monitoring was performed for manool tests at different doses (10, 20 and 40 mg/kg). Concentration-response curves for manool were obtained in the aorta rings, with endothelium, pre-contracted with phenylephrine (Phe) after incubation with Nω-nitro-L-arginine methyl ester(L-NAME) or oxadiazole [4,3-a]quinoxalin-1-one (ODQ). Nitric oxide (NOx) plasma levels were measured by chemiluminescence assay.

**Results:**

After manool administration, BP was reduced in normotensive and hypertensive groups, and this effect was inhibited by L-NAME in hypertensive animals only in 10 mg/kg dose. Ex vivo manool promoted vasorelaxation, which was inhibited by L-NAME and ODQ incubation or endothelium removal. NOx plasma levels increased in the hypertensive group after manool administration. Manool elicits endothelium-dependent vascular relaxation in rat aorta mediated by the NO/cGMP signaling pathway and BP reduction, also by NOx plasma increase. These combined effects could be involved in modulating peripheral resistance, contributing to the antihypertensive effect of diterpene.

**Conclusion:**

These effects together could be involved in modulating peripheral resistance, contributing to the antihypertensive effect of diterpene.

## Introduction

Diterpenes is a broad class of chemical metabolites, which are widely distributed in the flora, with more than 12,000 known compounds.^1^^,^^2^ They can be divided into two types: specialized (secondary) metabolism diterpenes and general (primary) metabolism diterpenes. Secondary diterpenes can have functions in the ecological interactions of plants with other organisms and benefits in pharmaceuticals, perfumes, resins and other industrial bioproducts with great economic relevance.^1^^,^^2^ Several secondary metabolites, such as terpenes, phenolic acids, polyphenols, flavonoids and anthocyanins, have been reported from Salvia species. These species are seen as excellent sources of diterpenes.^3^ According to chemotaxonomic findings, manool was previously reported in the following Salvia species: *S. sclarea, S. pubescens, S. lavandulifolia, S. hypoleuca*, *S. miltiorrhizae*. It is also present in other species, such as *Pinuscaribaea (Pinaceae), Lourteigiastoechadifolia (Asteraceae)* and *Halocarpusbiformis (Podocarpaceae)*. However, manool is the main diterpene of the various species of Salvia, and it is found in higher concentration in *Salvia officinalis*.^4^

The biosynthesis of the isoprene structural units of a wide variety of terpenes, including diterpenes, occurs by the deoxyxylulose pathway. This pathway rises to two distinct products: isopentenyl diphosphate (IPP) and dimethylallyl diphosphate (DMAPP). More specifically, manool, whose chemical composition is C_20_H_34_O, is a bicyclic labdane diterpene. Its structure is based on a 2E, 6E, 10E-geranylgeranyl pyrophosphate (GGPP) carbon skeleton.^5^^–^^7^

The discovery of new substances with antihypertensive activity, with low cost and few adverse effects, is still desirable and important to clinical use.^8^ However, several difficulties are encountered for this purpose, such as the choice of experimental model, obtaining standardized extracts and the difficulty of obtaining, isolating and identifying the active substances.^9^^,^^10^ The option to conduct research, from the indication of plants used by communities, shortens the route of developing a new drug, as researchers have, before starting scientific studies, a hint of which biological activity this medication might present.^11^^,^^12^

Diterpenes, in particular, are among the primary compound links to cardiovascular properties, such as vasorelaxant, inotropic, diuretic and hypotensive activity. The vascular action exerted by these compounds appears to involve multiple mechanisms. Such mechanisms are either independent or endothelium-dependents, prostacyclin, and increased blocking of voltage-dependent calcium channels.^13^^–^^17^

As previously described in the literature review, manool — C_20_H_34_O — is a labdane-type diterpene, commonly found in various plant families, it is the main diterpene of several species of Salvia, and is present in higher concentrations in *Salvia officinalis* (Figure 1).^1^^,^^3^^,^^18^^,^^19^ It is a species of the family *Lamiaceae (Labiateae)*, originating in southern Europe. It presents a habit of herbaceous growth or small shrub; it is a perennial plant that flourishes in the Southern Hemisphere between August and December.^20^

**Figure 1:** Manool chemical structure.^10^^,^^11^

Li et al.^21^ found that although manool possesses cardiovascular activity that is still unknown, it must be considered a crucial factor to be investigated. Moreover, it can be seen as a new driver for the treatment of heart disease and deserves further research.^4^^,^^21^^,^^22^ The experimental protocol included observations on plasma levels of nitric oxide (NO) in hypertensive animals and the impact of manool on the BP of animals following the administration of different doses of the compound.

Knowing that manool belongs to the class of diterpene compounds, with potential use in the treatment of hypertension, the present investigation was designed to assess the possible vasodilator effect and the cellular mechanisms involved in the relaxation response of aortic rings of rats. Therefore, the aim was to evaluate the in vivo hypotensive effect of manool and check the *ex vivo* vasorelaxation effect in aortic rings of rats.

## Material and Methods

### Ethics Statement and Animals

Animal handling policies and experimental procedures were reviewed and approved by the Institutional Committee for Animal Care from Faculdade de Medicina de Ribeirão Preto, Universidade de São Paulo (n. 060/210), following the directions of the European Commission's Directive 2010/63/EU. Thirty-four male Wistar rats (180–220 g) were housed under standard laboratory conditions (12 h light/dark cycle at 21 °C) with water and food ad libitum. The animals were allocated randomly into five groups of 7 animals for normotensive and hypertensive blood pressure protocols (normotensive vehicle, normotensive manool; hypertensive vehicle, hypertensive manool and hypertensive manool + L-NAME). The animals allocated to the normotensive groups were sham-operated, while animals allocated to the hypertensive groups underwent the surgical procedure 2K1C (two-kidney-one-clip hypertensive rats) for hypertensive induction. Another group of 6 animals that did not undergo any procedure (intact) was used for *ex vivo* vascular reactivity studies.

### Drugs

Manool, acetylcholine (ACh), 1H-[1,2,4]oxadiazole[4,3-a]quinoxalin-1-one (ODQ), and phenylephrine (Phe) were from Sigma Chemical Company (St. Louis, MO, USA); Nω-nitro-L-arginine methyl ester (L-NAME) was obtained from Calbiochem (San Diego, CA, USA); Vetec Química Fina Ltda furnished isoflurane from Abbott and all the salts used for Krebs solution preparation. Almost all the drugs were prepared with distilled water, and manool was solubilized in dimethyl sulfoxide (50 uL) and diluted in ethanol/water (2:10, total volume 200 uL). For vascular reactivity experiments, 100 uL was diluted in 900 uL of water, making the stock (10^−3^). From this stock, the curve was prepared. The volume used from this curve was 10 uL in a 10 ml cube. Therefore, after so many dilutions, the vehicle does not promote any effect on vascular reactivity.

### Induction of Hypertension

After i.p. anesthesia with ketamine (50 mg/kg) and xylazine (10 mg/kg), the renal artery was exposed. The hypertensive groups had partial constriction of the main left renal artery with a silver clip of 0.10 mm gap (2K1C), while the normotensive groups had the main left renal artery isolated but did not receive the clip (sham). To monitor hypertension development, systolic blood pressure (SBP) was noninvasively measured using a tail-cuff, once a week. (Kent Scientific Corporation, Connecticut, USA). The 2K1C rats were considered hypertensive with tail SBP ≥ 160 mmHg at the 3^rd^ week after the surgical procedures. The 2K1C rats with SBP < 160 mmHg at the 3^rd^ week were euthanatized. Less than 10% of animals had SBP < 160 mmHg. The sham-operated rats were included in the normotensive group.

### Manool Effect on Systolic Blood Pressure

Three weeks after hypertension induction, the animals were anaesthetized, and the femoral artery and vein were respectively cannulated for continuous measurement of systolic blood pressure (SBP) and drugs administration. After anesthesia (urethane, 2 mg/kg, intraperitoneal), vascular cannulation and stabilization period (20 minutes) with continuous real-time SBP recording, three doses of manool (10, 20 and 40 mg/kg) or vehicle (Dimethyl sulfoxide — DMSO — and water+ethanol) were administered to the normotensive and hypertensive rats. Each dose was given in a 200 *µ*L intravenous bolus, and the interval between each consecutive dose was 6 minutes. The animals that received vehicle did not receive manool. For each animal, the variation in systolic blood pressure (ΔSBP) was calculated subtracting the mean of the lowest SBP values immediately after manool administration from the average of the baseline SBP values before manool or vehicle bolus. Mean blood pressure was measured using MP System 100 A (BioPac System, Inc., Santa Barbara, CA, USA).

### Vascular Reactivity

Experiments were conducted in aortic rings from normotensive rats. Six male Wistar rats (280–300 g) were anaesthetized with inhalational isoflurane, followed by abdominal aorta exsanguinations and thoracotomy for thoracic aorta harvesting. The thoracic aorta was carefully dissected, confirmed to be free of connective tissue, and immediately immersed in Krebs solution. The Krebs solution was composed of NaCl (118.0 mM), KCl (4.7 mM), CaCl2 (2.5 mM), KH2PO4 (1.2 mM), MgSO4 (1.66 mM), glucose (11.1 mM), and NaHCO3 (25.0 mM); the solution had pH 7.4. The thoracic aorta immersed in Krebs solution was cut into rings that were 4–5 mm in length. For tests, the endothelium-denuded ring was removed by gently rubbing the internal surface vessel with a thin steel rod. This procedure effectively removes the endothelium, but it does not affect the ability of the vascular smooth muscle to contract or relax. The aortic rings were placed in 10 mL isolated organ bath containing Krebs solution, at 37 °C, and 95% O_2_/5% CO_2_ (pH 7.4) to measure the isometric force with Grass FT03 equipment (Grass Instrument Company, Quincy, MA, USA). Each ring was stretched to the optimal 2.0 g length-tension, determined in a pilot study, and was allowed to equilibrate for 60 min. During this time, tissues were washed every 15 min. The endothelium was considered to be present (E+) recording the Ach-induced 80% relaxation (10^−6^ M) after pre-contraction with Phe (10^−7^ M). Endothelium was considered absent (E−) when the relaxation response did not occur. Next, each ring was washed and re-equilibrated for 30 min. The aortic rings were precontracted with Phe (10^−7^ M) after a stable plateau was reached, and dose-response curves of manool were obtained. The concentration-response assays in the organ baths were carried out in the presence or absence of: L-NAME (2x10^−4^ M), a nonspecific nitric oxide synthase inhibitor and ODQ (10^−4^ M), a guanylyl cyclase inhibitor.^20^ The preparations were incubated with the inhibitors for 30 min. We did not perform dose-response curves with a vehicle because the dilution was performed in water. The initial solution 1 M (50 uL DMSO + 30 uL ethanol + 120 uL water) suffered serial dilution for 10^−1^ M in water.

### Indirect Plasma Measurements of NO

Blood samples (1 ml) were collected from the femoral artery, after the last dose-response curve from a normotensive vehicle and hypertensive manool, and placed in heparinized tubes. After blood centrifugation (3000×g, 10 minutes, 4 °C), the plasma was immediately immersed in liquid nitrogen and kept at −70 °C until nitrite and nitrate (NOx) measurements. Samples were analyzed in duplicates for NOx by ozone-based chemiluminescence assay. The plasma samples were briefly treated with cold ethanol (1 volume of plasma: 2 volumes of ethanol for 30 minutes at −20 °C) and centrifuged (4000×g, 10 minutes). The NOx levels were measured by injecting 25 μL of the supernatant in a glass purge vessel containing 0.8% of vanadium (III) in HCl (1 N) at 90 °C, which reduces NOx to NO gas. A nitrogen stream was bubbled through the purge vessel containing vanadium (III), then through NaOH (1 N), and then into a NO analyzer (Sievers® Nitric Oxide Analyzer 280, GE Analytical Instruments, Boulder, CO, USA).

### Statistical Analysis

The data are presented as mean ± standard error of the mean (SEM). We performed statistical analyses with Student's T-test, one-way (ANOVA), Bonferroni post-test and two-way repeated-measures of variance (ANOVA) with the Bonferroni post-test to detect potential differences between the values in the study. For each figure, the legend describes which test was performed for analysis. P<0.05 was considered significant (Prism 5.0, GraphPad Software, San Diego, CA, USA). A sample size of (N = 5–7) per group provided 95% power with a 0.05% significance level in protocols of in vivo blood pressure measurement. Moreover, a sample size of (N = 6–8) animals per group provided 95% power with a 0.05 significance level to detect a relative 10% reduction in the maximal contraction in precontracted vessels. The number of animals was based on the literature.^20^^,^^23^^,^^24^

## Results

Before surgical procedures, there were no differences in the BP between normotensive and hypertensive groups. However, after hypertension induction, from the 1^st^ to the 3^rd^ week, the BP was significantly higher in the hypertensive rats (130,6 mmHg versus 193,0 mmHg) (Figure 2).

**Figure 2:** Temporal evolution of systolic blood pressure (SBP) non-invasively in normotensive and hypertensive animals. The values represent mean ± standard error of mean arterial pressure before the clip placement surgery (pre-operative) and at three weeks following the surgery. * p<0.05 and # p <0.01 indicate a significant difference between the hypertensive group and the normotensive group. Two-Way ANOVA, Bonferroni post-test. n=14 normotensive and n=14 hypertensive.

The evaluation of body weight showed that, in the first week, the groups had similar loads. However, at the end of three weeks, the hypertensive group showed significantly lower values compared to the normotensive group (Table 1).

**Table 1:** Time evolution of body weight normotensive and hypertensive animals

Evolution of body weight (g)		
Groups	Initial	Final
Normotensive	233.4±7.1	480.2±10.2
Hypertensive	239.4±7.7	404.8±18.2*

Each value represents mean ± SEM.

* p<0.05 indicates significant difference between the hypertensive group and the normotensive group. Student's T-test.

In the *in vivo* SBP analysis, only the surgery (2K1C) was able to change the blood (normotensive vehicle versus hypertensive vehicle). Manool promoted a dose-dependent response on SBP, reducing the pressure significantly from the dose of 20 mg/kg in the normotensive group, and there is not any difference between 20 and 40 mg/kg in this group for manool. In the hypertensive group, only a lower dose of manool (10 mg/kg) reduced the SBP compared to the control (hypertensive vehicle) group, and the previous administration of L-NAME prevented the manool effect. In the hypertensive group, the manool effect was not dose-dependent (Figure 3).

**Figure 3:** Change in systolic blood pressure (ΔSBP) after administration of manool or vehicle in normotensive and hypertensive rats. Data are presented as mean ± standard error of the mean. Normotensive vehicle (n=7), normotensive manool (n=7), hypertensive vehicle (n=7), hypertensive manool (n=7) and hypertensive manool + L-NAME (n=7), * p< 0.05, ** p <0.01 indicates significant difference. Two-way ANOVA, Bonferroni post-test.

The plasma NOx is a little high in the normotensive group after manool administration, but it is not significant. However, in the hypertensive group, manool promoted an increase in plasma NOx levels (Figure 4).

**Figure 4:** Nitrite and nitrate levels (NOx) plasma in normotensive vehicle and manool and hypertensive vehicle and manool animals. One-way ANOVA, Bonferroni post-test (n=7). *p<0.01 indicates significant difference between hypertensive vehicle and hypertensive manool.

About vascular reactivity experiments, manool promoted a dose-dependent relaxation only in intact rings (Figure 5), precontracted with Phe. Incubation with either L-NAME or ODQ blocked the relaxation induced by manool in endothelium-intact rings in the same way of endothelium removal (Figures 6A and 6B).

**Figure 5:** Relaxation curve in endothelium-intact and endothelium-denuded rat thoracic aortic rings exposed to manool. The rings were pre-contracted with phenylephrine (Phe) (10-7.M). All values correspond to the mean ± SEM (n = 6). * p<0.05 and # p <0.001. Two-way repeated-measures ANOVA and Bonferroni post-test.

**Figure 6:** Relaxation curve in endothelium-intact rat thoracic aortic rings exposed to manool in the presence and absence of L-NAME (2x10-4 M) or oxadiazole [4,3-a]quinoxalin-1-one (ODQ) (10-4 M). (A) dose-response curve and (B) Bar graph Emax. The rings were pre-contracted with phenylephrine (Phe) (10-7.M). All values correspond to the mean ± SEM (n = 6). *p< 0.05 and # p< 0.001 indicate significant differences between each group and the control group (vessels with endothelium); Two-way repeated-measures ANOVA and Bonferroni post-test.

## Discussion

Previous research has shown that labdane diterpenes have a wide range of pharmacological effects, such as the ability to inhibit HIV replication, prevent common colds, it is antimalarial, antibacterial, anti-inflammatory, antihyperglycemic, prevents dysentery, besides suppressing various cancerous cells.^6^^,^^13^ On the cardiovascular side, they showed: significant reduction of stenosis in atherosclerotic arteries, associated with the fewer restenosis rates after angioplasty in rabbits; reduction of *ex vivo* platelet aggregation, and antihypertensive action in rats.^13^^,^^15^^–^^17^^,^^25^ They are thus seen as a promising source of new prototypes for the discovery and development of new agents of cardiovascular therapeutics.

Diterpenes, in particular, are among the significant compounds with binding to cardiovascular properties, such as vasorelaxant, inotropic, diuretic and hypotensive activity.^26^ The vascular action exerted by these compounds seems to involve multiple mechanisms, such as dependent and independent endothelium, increase of prostacyclin and blockade of voltage-dependent calcium channels.

In the present study, we used the 2K1C model for investigating the possible antihypertensive effect of manool. This model produced satisfactory results, for hypertension induction, with a significant increase in blood pressure in animals, after three weeks of surgery. Even the first week post-surgery, the 2K1C SBP was higher than in a normotensive animal. SBP found in hypertensive animals agree with other authors who evaluated a similar model.^23^^,^^27^^,^^28^

The results obtained after administration of 3 increasing doses of manool showed that this compound was able to reduce BP in both normotensive and hypertensive rats. In normotensive animals, manool presents a positive dose-response effect. This finding is different from other natural compounds, including Rosmarinic acid, which reduced BP only in hypertensive animals.^23^ This response profile is not observed in hypertensive animals, where the dose increase does not represent a more significant effect. ΔSBP is the same after 10, 20 and 40 mg/kg of manool in hypertensive animals; in other words, regardless of the doses, maximum blood pressure was about 40–50 mmHg. However, as in the hypertensive vehicle group there was a reduced SBP, only the 10 mg/kg was able to effectively reduce the pressure.

Our hypothesis to this antihypertensive effect of manool was based on recent studies about the vasodilator activity of diterpenes mediated by NO.^13^^,^^15^^,^^16^^,^^26^ It has been demonstrated that hypertension has a strong association with the formation of reactive oxygen species (ROS).^29^ Consequently, inactivation of NO by superoxide induces the development of endothelial dysfunction in cardiovascular diseases.^30^ The property of some compounds to increase NO can be attractive to reducing the endothelial dysfunction of hypertension. Our findings indicate that the antihypertensive effect of manool can be partially mediated by NO once L-NAME administration before manool injection blocks SBP reduction in hypertensive animals only at a dose of 10 mg/kg. Corroborating these findings, the plasma NOx concentration was increased significantly only in the hypertensive animals that received manool. Some NOx studies in the 2K1C model show that hypertension can reduce these levels, but our finding is in disagreement with this data, perhaps because of the time of 2K1C surgery.^31^^,^^32^ Though the full antihypertensive effect of manool remains unknown, other hypotheses can be raised, such as ACE (angiotensin-converting enzyme) inhibition and modulation.^33^ It was demonstrated that, in the 2K1C model, there is an increase in plasma ACE activity and some natural peptides from rice, terpenes, phytoestrogen and polyphenol compounds can reduce this ACE activity,^20^^,^^34^^,^^35^ which could characterize this mechanism as complementary to NO on BP maintenance.

It would be possible to attribute the antihypertensive effect of manool to a direct effect on vascular reactivity that does not include an increase of systemic NO. The present study showed that manool induces relaxation in rat aorta only in the presence of endothelium and pre-incubation of the aortic rings with nitric oxide synthase (NOS) or guanylyl cyclase (GC) inhibitors. The cardiovascular properties of diterpene are related to Ca^2+^ channels blockade and NO/cGMP (cyclic guanosine monophosphate) activation.^13^ The endothelium produces potent vasodilators, such as the endothelium-derived relaxing factor (EDRF, NO), prostacyclin, and endothelium-derived hyperpolarizing factor (EDHF). NO is the predominant mediator in conductance and large arteries, whereas EDHF and prostacyclin are more prevalent in smaller arteries, such as the mesenteric vessels, coronary arteries and peripheral resistance vessels.^36^ Corroborating our findings, some diterpenes, such as 14-deoxy-11,12-dihydroandrographolide and 14-deoxyandrographolide have been reported to dilate aortic rings. The compound 14-deoxy-11,12-dihydroandrographolide had a hypotensive effect in anaesthetized rats. Both compounds exert their vasorelaxant activity by the release of NO and activation of the guanylate cyclase pathway, as well as the blockade of Ca^2+^ influx through both voltage- and receptor-operated Ca2+ channels.^13^^,^^37^^–^^39^ In the present study, we also suggest that manool has an endothelium-dependent vasorelaxant effect operating via the NO/cGMP pathway.

## Conclusion

In summary, manool elicits endothelium-dependent vascular relaxation in rat aorta mediated by the NO/cGMP signaling pathway and BP reduction also by NOx plasma increase. These effects together could be involved in modulating the peripheral resistance, contributing to the antihypertensive effect of this diterpene.
